# Perspective: the potential of wastewater-based surveillance as an economically feasible game changer in reducing the global burden of pediatric respiratory syncytial virus infection

**DOI:** 10.3389/fpubh.2023.1316531

**Published:** 2024-01-12

**Authors:** Nisha Thampi, Elisabeth Mercier, Bosco Paes, James O. Edwards, Barry Rodgers-Gray, Robert Delatolla

**Affiliations:** ^1^Department of Pediatrics, Children’s Hospital of Eastern Ontario, Ottawa, ON, Canada; ^2^Department of Pediatrics, University of Ottawa, Ottawa, ON, Canada; ^3^Department of Civil Engineering, University of Ottawa, Ottawa, ON, Canada; ^4^Department of Pediatrics (Neonatal Division), McMaster University, Hamilton, ON, Canada; ^5^Violicom Medical Limited, Aldermaston, United Kingdom

**Keywords:** respiratory syncytial virus, wastewater-based surveillance, economics, pediatric hospitalization, community incidence, season start date, passive immunoprophylaxis, hospital level preparedness

## Abstract

Respiratory syncytial virus (RSV) is the leading viral cause of childhood bronchiolitis and pneumonia causing over 3 million hospitalizations and 100,000 deaths in children under 5 years of age annually. Wastewater-based surveillance (WBS) has proven an effective early warning system for high-consequence pathogens, including SARS-CoV-2, polio, mpox, and influenza, but has yet to be fully leveraged for RSV surveillance. A model predicated on the Canadian province of Ontario demonstrates that implementation of a WBS system can potentially result in significant cost savings and clinical benefits when guiding an RSV preventive program with a long-acting monoclonal antibody. A network of integrated WBS initiatives offers the opportunity to help minimize the devastating global burden of RSV in children by optimizing the timing of preventive measures and we strongly advocate that its benefits continue to be explored.

## 1 Introduction

The benefits of a global, integrated wastewater-based surveillance (WBS) system was recently reported to provide an early warning of high-consequence pathogens, such as SARS-CoV-2, polio, mpox, and influenza (1). We unreservedly agree with this proposal and strongly advocate for respiratory syncytial virus (RSV) to be included as a priority pathogen in the evaluation of integrated WBS systems.

The World Health Organization has long recognized severe RSV infection as a major health care burden, causing greater than 3 million RSV related hospitalizations (RSVHs) and 100,000 deaths every year in children aged less than 5 years as well as a substantial burden of illness in older adults, greater than 60 years of age (2–4). Until recently, the options to prevent severe RSV infection have been limited to basic hygiene measures and selective prophylaxis of the most vulnerable pediatric populations with the short-acting monoclonal antibody, palivizumab. Newer preventive options that include long-acting monoclonal antibodies, such as nirsevimab, and maternal, child and adult vaccines (5), have the potential to profoundly impact the global burden of RSV. Accurate knowledge on the timing of community surges in RSV infection is essential to guide the planning, implementation, and real-time assessment of prophylaxis and vaccine programs, thereby ensuring maximum coverage and protection against RSV.

A Canadian study undertaken in Ontario recently reported that RSV-WBS resulted in a 12-day lead time in pediatric RSVH surge and 36-day lead time in defining the start to the pediatric RSV season vs. clinical surveillance alone (CS) (6). These results suggest that WBS may provide an important, population-level, early warning signal to prime policy decision-makers to plan and implement timely RSV prevention strategies.

## 2 Clinical and economic benefits of wastewater-based surveillance

**Figure 1:**
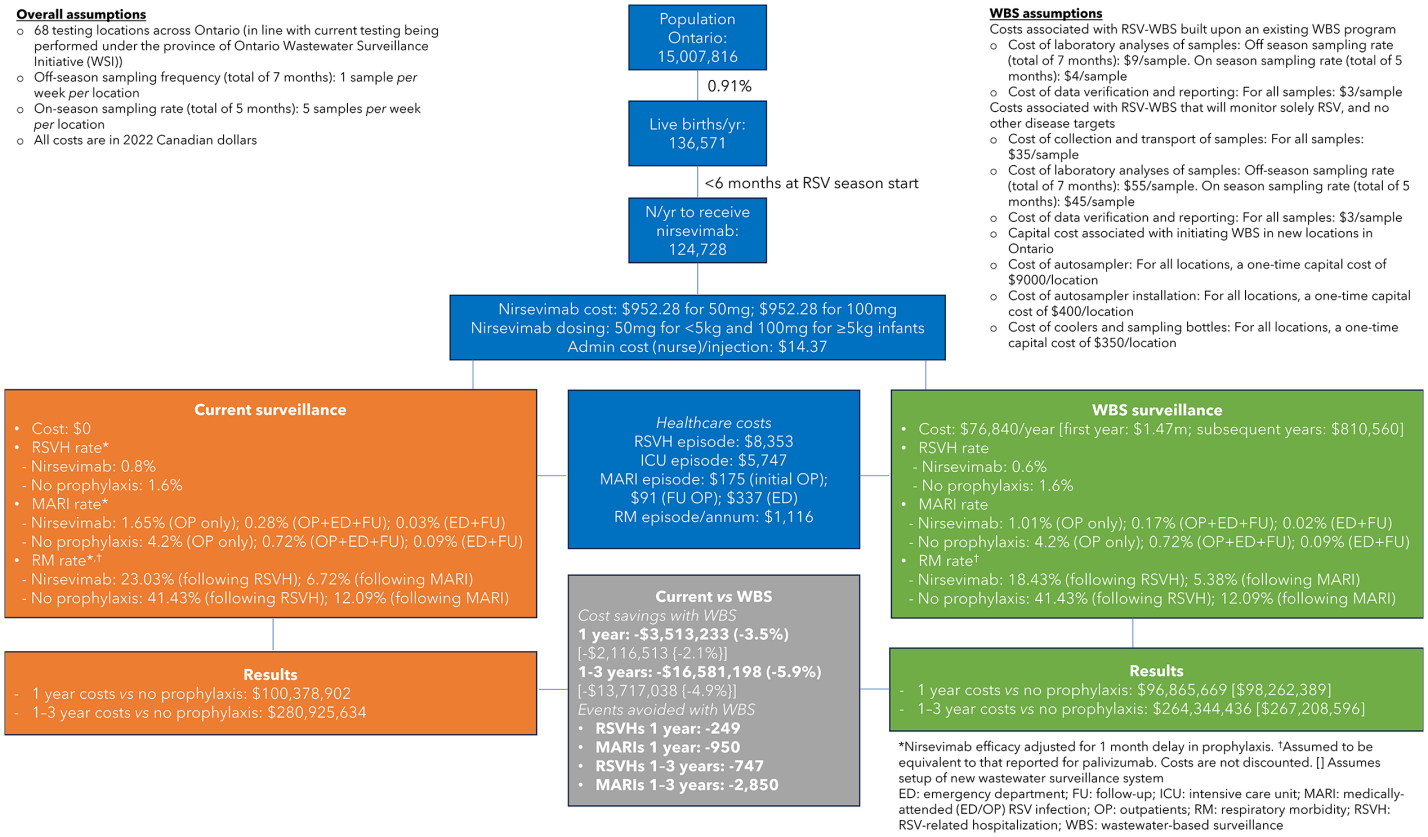
Cost-consequence model comparing wastewater-based surveillance (WBS)- vs. clinical surveillance (CS)-guided respiratory syncytial virus (RSV) prophylaxis programs in Ontario, Canada.

To explore the potential impact and costs associated with WBS, we developed a cost-consequence model that compared WBS- vs. CS-guided nirsevimab prophylaxis programs for infants in Ontario aged <6 months at the start of the RSV season (Figure 1), where RSV epidemics were traditionally seasonal (November to March) in the pre-pandemic COVID-19 era. Currently, the start of the RSV season is determined by CS, typically defined as the first 2 consecutive weeks when >10% of the total samples tested for respiratory pathogens are positive for RSV (7). Similarly, the end of the season is defined when the proportion of RSV-positive tests is <10% for 2 consecutive weeks (7). Testing for RSV infection primarily occurs among children requiring hospitalization, which inherently reflects a lag in determining the community incidence of RSV. This form of CS also relies on timely submission of test results to public health agencies that will determine the start and end of the pediatric RSV season and initiation of immunoprophylaxis.

Nirsevimab was chosen for our cost analysis as it is indicated for all infants <8 months of age at the start of the RSV season (8–10) and could have a dramatic impact on reducing the intensity of RSV epidemic waves and concomitant capacity surges on pediatric acute care systems. Based on the Canadian data, we assumed that the RSV season was detected 1 month earlier using WBS than CS and, in both scenarios, spanned 5 months (6). Nirsevimab efficacy in the CS scenario was adjusted by having 1 month without prophylaxis to account for the earlier implementation of the RSV prophylaxis program with WBS. For example, the RSVH rate was 0.6% for WBS based on the nirsevimab registrational trial (9) vs. 0.8 and 1.6% for CS in infants who, respectively, did or did not receive nirsevimab. Efficacy rates for nirsevimab in the prevention of medically-attended emergency room/outpatient RSV infections (MARI) and subsequent respiratory morbidity were similarly adjusted.

It was assumed, based on current WBS systems, that off-season sampling would take place at a frequency of one sample *per* week *per* location (CAN 9/sample) for a total of 7 months, increasing to five samples *per* week (CAN 4/sample) during the RSV season, across 68 testing locations in Ontario. When RSV testing was added to an existing WBS system, we calculated that the costs incurred would be CAN 76,840 in the first year, which increased to CAN 1.47 m when considering the capital costs associated with setting-up a new provincial WBS system. Once established, the cost of a new system decreased to CAN 810,560 in subsequent years. No additional costs were assumed for current CS. Other costs included in the analysis were for nirsevimab (infants <5 kg: CAN 952.28 for 50 mg; infants ≥5 kg: CAN 952.28 for 100 mg), RSVHs (CAN 8,353/episode), intensive care unit stays (CAN 5,747/episode), MARIs (CAN 175–$337/episode), and respiratory morbidity (CAN 1,116/annum/episode) (11), all of which were equivalent for both WBS and CS. Costs in the model were calculated based on 136,571 annual live births in Ontario, of which 124,728 infants were aged <6 months at the start of the RSV season and thereby eligible for prophylaxis.

The results of the model found that WBS was associated with savings of CAN 2.1–3.5 m in the first year and CAN 13.7–16.6 m over 3 years, with larger savings accrued when RSV testing was added to an existing WBS system. These savings translate into a benefit–cost ratio of 45.7 in the first year and 71.9 over 3 years, when RSV is added to an existing WBS system. This implies that for every CAN 1 spent on RSV-WBS, there is CAN 45.7–71.9 return on investment. The corresponding ratios for a new RSV-WBS are 1.4 for year 1 and 4.4 over 3 years (CAN 1.4–4.4 return for every CAN 1 invested). Importantly, WBS-guided prophylaxis resulted in 249 fewer RSVHs and 950 fewer MARIs *per* year vs. CS-guided prophylaxis.

In a sensitivity analysis, the advantage of WBS over CS was shortened to a 2-week earlier detection of the RSV season, which resulted in a saving of CAN 321 k–1.7 m in the first year and CAN 5.3–8.2 m over 3 years. Under this scenario, WBS-guided prophylaxis resulted in 125 fewer RSVHs and 475 fewer MARIs *per* year versus CS-guided prophylaxis.

## 3 Discussion

This simple model, covering one Canadian province and one preventive strategy, highlights the substantial benefit that WBS can confer in this new era of RSV prevention once the strategy is proven to be reproducible and valid throughout sequential RSV seasons and generalizable across communities. A fully integrated and publicly-funded network of WBS initiatives across high-, middle-, and low-income countries, affords a real opportunity for policy decision makers and public health agencies to intervene early and substantially reduce the impact on already strained pediatric bed capacities and associated hospital costs at the onset of the RSV season. WBS also provides a unique opportunity to minimize the devastating global burden of RSV in children by optimizing the timing of preventive measures.

## Data availability statement

The original contributions presented in the study are included in the article/supplementary material, further inquiries can be directed to the corresponding author.

## Author contributions

NT: Conceptualization, Writing – original draft, Writing – review & editing. EM: Conceptualization, Writing – original draft, Writing – review & editing. BP: Conceptualization, Writing – original draft, Writing – review & editing. JE: Conceptualization, Formal Analysis, Writing – original draft, Writing – review & editing. BR-G: Conceptualization, Formal Analysis, Writing – original draft, Writing – review & editing. RD: Conceptualization, Writing – original draft, Writing – review & editing.
